# Practical Notes on Diagnosis and Treatment

**Published:** 1912-11-23

**Authors:** 


### PRACTICAL NOTES ON DIAGNOSIS AND TREATMENT.

**Graves's Disease in Children.**

THIS is not common, but it certainly occurs; quite decided cases have been seen at four to five years of age.

**Salvarsan in Pernicious Anæmia.**

DR. BYROM BRAMWELL, to whom we owe the use of arsenic in the treatment of pernicious anæmia, has recently reported some cases of the disease healed with excellent results by salvarsan.

**Salt in Epilepsy.**

BROMIDE of potassium is often much more efficacious in epilepsy if the patient is deprived of salt. Three pints of milk daily, vegetables, carbo-hydrates, and salt-free bread form a suitable diet list.

**Enuresis in Females.**

IN obstinate cases success may follow the application of silver nitrate solution (20 to 40 grains to the ounce) to the urethra. The application may be renewed on two or three occasions at intervals of a few days.

**Sodium Salicylate.**

THOUGH widely used and generally beneficial this drug does sometimes produce troublesome effects. Apart from deafness, etc., the use of the salicylate has been followed by hæmaturia in children, and in some few instances adults have become blind when taking it in large doses.

**Knee-Jerks in Pneumonia.**

THE knee-jerks are often absent in croupous pneumonia in children, and the defect may be observed prior to the appearance of the physical signs. Such absence does not occur in lobular pneumonia nor in the early stages of meningitis; it may, therefore, prove of diagnostic importance.

**Diachylon Poisoning.**

THE practice of taking pills of diachylon plaster to produce abortion is not uncommon; it is the explanation of not a few cases of ill-defined illness in women. In these cases the picture of lead-poisoning is often an incomplete one, such a symptom as wrist-drop, for example, being quite unusual.

**Temperature in Hepatic Disease.**

IN cases of impacted gall-stone, well-marked, though short-lived febrile attacks are not uncommon, and they may be associated with rigors. These are generally recognised. It is, however, less well known that similar attacks, or a more prolonged pyrexia, may occur in cancer of the liver, and much the same is true of cirrhosis.

**Olive Oil in Constipation.**

BOTH in colitis and in cases of atony of the bowel leading to constipation olive oil by the rectum is often useful. Some five to ten ounces should be introduced gradually from a funnel or tube; a syringe should not be used. The oil is administered at bedtime and is allowed to remain until the bowels act on the following morning.

**Dose of Digitalis.**

MACKENZIE'S work has shown that it is particularly in cases of mitral disease associated with auricular fibrillation that digitalis is successful. The dose must be accommodated to the individual patient; an amount far beyond the conventional limit is often found to be necessary. On the whole, there is no preparation so reliable as a well-prepared tincture.

**Tubercular Meningitis.**

A CASE has been reported in which recovery followed the use of an iodoform ointment (1 in 10) to the shaved scalp. The ointment was applied four times a day, and after each application the scalp was covered with an oil-silk cap. The iodoform produced much local irritation, swelling of the eyelids, etc., but the patient, whose symptoms included stupor, convulsions, squint, etc., made a complete recovery.

**Intermittent Claudication.**

THIS term is applied to the condition in which a patient has no complaint so long as he is sitting still, but after walking a short distance he suffers pain, numbness, or cramp in his legs. It has been called "angina pectoris of the legs." The underlying cause is supposed to be sclerosis and spasm of the arteries, and the pulse may be absent from the dorsalis pedis and posterior tibial vessels. Abstinence from tobacco, a vegetable diet, and iodides form the treatment, but many of the cases are very obstinate.

**Psoriasis.**

WHILST in the chronic forms the administration of arsenic may be helpful, this drug is altogether unsuitable in acute and rapidly spreading cases of the disease. Here the indication is for the use of antimony—say, 5 to 10 minims of the B.P. vinum. One of the best local applications in chronic cases is hydrarg. ammon., acid chrysophanic, aa. grs. x, liq. carb. deterg. ♏x, adeps benz., ℥j. To be successful with this the patches should first be scrubbed with soap and water, and the ointment then rubbed in vigorously and patiently.

**Ocular Paralyses.**

THESE occurring in middle life always demand a suspicion of commencing degenerative changes in the central nervous system; they are not very infrequently the first clinical sign of tabes dorsalis, and in such circumstances the paralysis may be slight and of brief duration. No doubt ocular palsies are sometimes the result of "cold" or "rheumatism," but until the lapse of time shows the interpretation to be a "simple" one the suspicion above suggested must always exist; and if there is a history of syphilis this suspicion is much strengthened.

